# Supplementary material for: Validation and Psychometric Properties of the Spanish Version of the Hopkins Symptom Checklist-25 Scale for Depression Detection in Primary Care
Source: Int J Environ Res Public Health. 2021 Jul 24;18(15):7843. doi: 10.3390/ijerph18157843 (PMC8345472; doi:10.3390/ijerph18157843)
Supplement: Supplementary file 1 [file ijerph-18-07843-s001.zip › ijerph-1277424-supplementary.pdf]

Table S1. Participation and non-response patterns.

Number of Missing Items	Missing for Which Variables?	*n* (%)
25	12345 67890 12345 67890 12345	22 (2.78)
13	_234_ __8_0 123_5 ____0 12__5	1 (0.13)
3	_23__ _____ _____ 6____ _____	1 (0.13)
1	_2___ _____ _____ _____ _____	2 (0.25)
5	__3__ 6_8__ _____ __8__ 1____	1 (0.13)
5	____5 6789_ _____ _____ _____	1 (0.13)
3	____5 6____ _____ __8__ _____	1 (0.13)
2	____5 _____ _____ ___9_ _____	1 (0.13)
2	_____ _7___ _____ _7___ _____	1 (0.13)
1	_____ _7___ _____ _____ _____	3 (0.38)
1	_____ __8__ _____ _____ _____	1 (0.13)
1	_____ ___9_ _____ _____ _____	2 (0.25)
2	_____ _____ 1____ _____ 1____	1 (0.13)
1	_____ _____ _2___ _____ _____	1 (0.13)
2	_____ _____ __34_ _____ _____	1 (0.13)
1	_____ _____ __3__ _____ _____	2 (0.25)
2	_____ _____ ___4_ _7___ _____	1 (0.13)
1	_____ _____ ___4_ _____ _____	2 (0.25)
2	_____ _____ _____ 67___ _____	1 (0.13)
2	_____ _____ _____ 6____ 1____	1 (0.13)
1	_____ _____ _____ 6____ _____	1 (0.13)
1	_____ _____ _____ __8__ _____	1 (0.13)
2	_____ _____ _____ ___90 _____	1 (0.13)
1	_____ _____ _____ ____0 _____	1 (0.13)
2	_____ _____ _____ _____ 1__4_	1 (0.13)
1	_____ _____ _____ _____ 1____	1 (0.13)
0	_____ _____ _____ _____ _____	737 (93.29)
	Total	790 (100)

The first column refers to how many items are unanswered. The second one specifies which items are left as missing, ordered by units and dozens. Finally, the far right column shows how many participants performed the concrete non-response pattern, e.g. 3 participants left item 7 as a missing value

Table S2. Correlation among items.

	Item 1	Item 2	Item 3	Item 4	Item 5	Item 6	Item 7	Item 8	Item 9	Item 10	Item 11	Item 12	Item 13	Item 14	Item 15	Item 16	Item 17	Item 18	Item 19	Item 20	Item 21	Item 22	Item 23	Item 24	Item 25
Item 1	1.000																								
Item 2	0.538	1.000																							
Item 3	0.251	0.270	1.000																						
Item 4	0.349	0.385	0.393	1.000																					
Item 5	0.294	0.284	0.360	0.410	1.000																				
Item 6	0.188	0.223	0.340	0.372	0.435	1.000																			
Item 7	0.288	0.364	0.445	0.612	0.397	0.296	1.000																		
Item 8	0.165	0.193	0.303	0.308	0.261	0.193	0.320	1.000																	
Item 9	0.318	0.416	0.166	0.316	0.208	0.277	0.274	0.174	1.000																
Item 10	0.298	0.382	0.352	0.561	0.403	0.330	0.539	0.215	0.307	1.000															
Item 11	0.231	0.286	0.588	0.444	0.402	0.358	0.456	0.315	0.194	0.419	1.000														
Item 12	0.322	0.340	0.297	0.367	0.302	0.182	0.373	0.131	0.271	0.371	0.356	1.000													
Item 13	0.308	0.317	0.236	0.350	0.230	0.245	0.312	0.229	0.231	0.363	0.308	0.317	1.000												
Item 14	0.267	0.253	0.299	0.307	0.244	0.183	0.310	0.191	0.137	0.257	0.386	0.279	0.266	1.000											
Item 15	0.207	0.296	0.373	0.365	0.257	0.211	0.366	0.125	0.198	0.378	0.392	0.405	0.342	0.354	1.000										
Item 16	0.226	0.290	0.277	0.341	0.209	0.109	0.364	0.189	0.248	0.331	0.323	0.374	0.281	0.377	0.528	1.000									
Item 17	0.332	0.395	0.471	0.508	0.378	0.307	0.536	0.353	0.301	0.528	0.519	0.461	0.469	0.441	0.614	0.577	1.000								
Item 18	0.181	0.239	0.186	0.246	0.186	0.287	0.295	0.089	0.326	0.215	0.244	0.264	0.212	0.150	0.319	0.324	0.378	1.000							
Item 19	0.230	0.312	0.307	0.326	0.303	0.177	0.382	0.185	0.225	0.381	0.350	0.372	0.250	0.285	0.465	0.519	0.518	0.306	1.000						
Item 20	0.312	0.306	0.288	0.466	0.319	0.240	0.461	0.207	0.210	0.458	0.371	0.379	0.304	0.278	0.317	0.310	0.459	0.198	0.310	1.000					

Item 21	0.239	0.262	0.328	0.338	0.292	0.154	0.336	0.193	0.182	0.363	0.432	0.358	0.282	0.349	0.479	0.493	0.560	0.289	0.377	0.311	1.000				
Item 22	0.251	0.302	0.547	0.409	0.335	0.274	0.434	0.236	0.159	0.367	0.621	0.375	0.274	0.394	0.433	0.413	0.534	0.231	0.406	0.372	0.519	1.000			
Item 23	0.173	0.252	0.304	0.243	0.183	0.226	0.313	0.148	0.216	0.237	0.340	0.335	0.244	0.286	0.414	0.370	0.435	0.323	0.402	0.333	0.389	0.451	1.000		
Item 24	0.092	0.151	0.220	0.215	0.183	0.222	0.139	0.128	0.191	0.191	0.184	0.150	0.158	0.191	0.175	0.195	0.202	0.146	0.190	0.248	0.204	0.209	0.158	1.000	
Item 25	0.222	0.239	0.275	0.369	0.324	0.199	0.357	0.335	0.158	0.304	0.344	0.251	0.288	0.226	0.293	0.304	0.379	0.187	0.251	0.287	0.246	0.293	0.253	0.208	1.000
